# Longitudinal Optical Coherence Tomography Measurement of Retinal Ganglion Cell and Nerve Fiber Layer to Assess Benign Course in Multiple Sclerosis

**DOI:** 10.3390/jcm12062240

**Published:** 2023-03-14

**Authors:** Abbas Al-Hawasi, Neil Lagali, Per Fagerholm, Yumin Huang-Link

**Affiliations:** 1 Division of Ophthalmology, Department of Biomedical and Clinical Sciences, Faculty of Medicine, Linköping University, 581 83 Linköping, Sweden; 2 Division of Neurology, Department of Biomedical and Clinical Sciences, Linköping University, 581 85 Linköping, Sweden

**Keywords:** retinal ganglion cell, retinal nerve fiber layer, ganglion cell complex, multiple sclerosis, benign multiple sclerosis, optical coherence tomography, biomarker, neural biomarker

## Abstract

A benign form of multiple sclerosis (BMS) is not easily diagnosed, but changes of the retinal ganglion cell layer-inner plexiform layer (GCL-IPL) and retinal nerve fiber layer (RNFL) may be sensitive to the disease. The aim of this study was to use optical coherence tomography (OCT) to investigate longitudinal changes of GCL-IPL and RNFL in BMS. Eighteen patients with BMS and 22 healthy control (HC) subjects were included, with a mean follow-up period of 32.1 months in BMS and 34.3 months in HC. Mean disease duration in BMS was 23.3 years, with 14 patients left untreated. Unilateral optic neuritis (ON) was found in eight patients. Non-ON eyes showed thinner GCL-IPL layer in the BMS group relative to HC (*p* < 0.001). The thinning rate of GCL-IPL in non-ON BMS, however, was −0.19 ± 0.15 µm/year vs. 0 ± 0.11 µm/year for HC (*p* = 0.573, age-adjusted). Thinning rate of RNFL in non-ON BMS was −0.2 ± 0.27 µm/year vs. −0.05 ± 0.3 µm/year for HC (*p* = 0.454, age adjusted). Conclusions: Thinning rate of the GCL-IPL and RNFL in BMS is similar to the healthy population but differs from the thinning rate in relapsing-remitting MS, presenting a non-invasive OCT-based criterion for assessing a benign course in multiple sclerosis.

## 1. Introduction

Multiple sclerosis (MS) is a chronic inflammatory disease that affects the central nervous system (CNS). The disease is characterized by the loss of myelin sheath around the nerves in the brain and spinal cord, leading to functional losses, including motor, sensation, and vision.

Benign MS (BMS) was considered as a subtype of MS [1] comprising up to 10% of all cases of MS (although this figure varies in the literature from 5–64%) [2] and is defined by only minimal disability over time [3]. The diagnostic criterion for BMS is based on the expanded disability status scale (EDSS) assessed over many years. BMS patients keep mobile with minimal disability (EDSS ≤ 2 at 10 years after the first signs of MS or EDSS ≤ 3 after 15 years) [4,5,6]. This group may not require long-term aggressive therapy as they are fully mobile and functioning, but monitoring is still required. The diagnosis of BMS can only be made retrospectively [7], and it is difficult to predict which patient will continue to follow a benign clinical trajectory after 10–15 years [8]. Prior studies showed the difficulty of differentiating between the BMS course and the progressive relapsing-remitting MS (RR-MS) based on a cerebrospinal fluid test [9] or neuroimaging [10]. Further, BMS course may transfer to RR-MS or secondary progressive MS (SP-MS) after many years with a silent course of MS [11,12].

The recent classification of MS does not consider BMS as a subtype; rather, it classifies MS into either RR-MS, including clinically isolated syndrome (CIS), or progressive disease [13,14]. The new recommendations of MS therapeutic strategy are to treat all RR-MS cases early, including CIS patients [11]. BMS with minimal disability and benign disease course will be treated even though there is no curative therapy for MS; current therapies aim at reducing the relapse risk and/or progression of the associated disability [11].

In recent years, the emerging modality of optical coherence tomography (OCT) examination of the ganglion cell layer-inner plexiform layer (GCL-IPL) and retinal nerve fiber layer (RNFL) has provided a non-invasive window to monitor MS course [15,16]. Not only thickness changes of retinal layers can be detected by OCT, but also changes in the reflectivity of different layers may be an early indicator of ultrastructural changes [17,18,19]. The RNFL was a main focus for OCT studies in different neurological diseases, including MS; the GCL-IPL was studied to a lesser extent. Most of the studies were cross-sectional studies, and very few describe longitudinal changes in RNFL; additionally, there is no OCT study presenting longitudinal changes in the GCL-IPL particularly in BMS. Previously, we reported OCT parameters, including GCL-IPL and RNFL, in a group of BMS patients compared to RR-MS and healthy controls [20]. In the current study, we report the results of the continued follow-up of this unique cohort of BMS patients. This study’s aim was to investigate the longitudinal changes of the retinal GCL-IPL and peripapillary RNFL layers during the natural course of largely untreated BMS, compared to a group of healthy controls. We hypothesized that OCT-based parameters could be used to follow the disease course and to identify a BMS subtype.

## 2. Material and Method

### 2.1. Subjects

This was a longitudinal, prospective study conducted between March 2013 and April 2019 with the enrollment of 20 patients with BMS. The diagnosis of clinically definitive MS was based on the 2010 revision of the McDonald Criteria [21]. The study was approved by the Regional Ethical Review Board in Linköping, Sweden (2013/1411-31). All patients were examined at least once with magnetic resonance imaging (MRI) of the brain and the spinal cord and had at least one lumbar puncture (LP) for the routine examination of the cerebrospinal fluid (CSF). BMS was defined as EDSS ≤ 2 after 10 years since MS diagnosis [4,5] or EDSS ≤ 3 after 15 years since diagnosis [6]. Following enrollment, all patients at their next routine visit to the Neurology Department, Linköping University Hospital, were examined with OCT, and this was considered the baseline visit. Patients with any ophthalmological disease that may have affected the OCT measurements, such as glaucoma, wet age-related macular degeneration, diabetic retinopathy, acute optic neuritis (ON), and high refractive errors, were excluded from the study. During the baseline visit, the EDSS score was evaluated. Clinical data, including EDSS, and therapy were revised at the final study visit. The time interval between the two study visits was based on the clinical need for follow-up and was not pre-determined for the study. Most of the patients had more than one visit between the baseline and final study visit as a routine clinical follow up for BMS.

A parallel healthy control group (HC) of 26 healthy subjects with no history and no symptoms of any neurological disease was recruited for the study during the same period. Exclusion criteria were the same as for the BMS group.

### 2.2. Optical Coherence Tomography (OCT) Examination

The Cirrus HD-OCT (model 4000, Carl Zeiss Meditech, software version 6.5) was used to examine retinal microstructures. The OCT examination was performed on two occasions for each study subject: at the study baseline visit and on a later occasion, which was considered as the final study visit. The peripapillary retinal nerve fiber layer (RNFL) was measured using the built-in 200 × 200 Optic Disc Cube protocol centered on the optic disc along 3.4 mm-diameter circles. The macular GCL-IPL was measured using the built-in 512 × 128 Macular Cube protocol within a 6 × 6 mm area centered on the fovea. Total retinal volume (TV) and the average macular thickness [22] were also measured using the Macular Cube protocol. The OCT examinations were repeated as needed until the most accurate examination was obtained with a signal strength of 7/10 or higher. The examinations were performed in a dark room without pharmacologic pupillary dilatation. The built in automated segmentation was used to measure the average RNFL and GCL-IPL thickness. The thinning rate of GCL-IPL and RNFL were calculated by dividing the difference between the baseline OCT measure and the final OCT measure by the duration between the measurements in years.

### 2.3. Statistical Analysis

All data were entered into an Excel spreadsheet (Microsoft Office, Microsoft Corp, Redmond, WA, USA) and then transferred into Statistical Package for the Social Sciences (SPSS) version 28 (SPSS, IBM Corp, Chicago, IL, USA). Data analyses were first performed using data from all measured eyes, and thereafter using only the eye without ON (in cases with ON) or a random eye (in cases without ON) for the BMS group. A single eye per subject from the HC group was chosen randomly. The results for group comparisons represent a single eye per subject chosen as above.

Descriptive statistics for the BMS and HC groups were generated using SPSS. The Shapiro–Wilk test was used to assess the normal distribution of the data. Independent *t*-tests were used to compare longitudinal variables across groups. Chi-Square tests were used for categorical variables. A univariate general linear model was used to compare the rate of thinning of the RNFL and GC-IPL between the two groups with adjustment for age and sex. A bivariate correlation test by Pearson’s correlation was used to assess for association between the thinning rate and disease duration. In all cases, a two-tailed alpha level of <0.05 was considered statistically significant.

## 3. Results

### 3.1. Demographic Characters of BMS and HC

Of the twenty patients in the BMS group, one patient had a stroke during the study and another patient discontinued the study; their data are not included. A total of 18 patients (11 females) with BMS on continuous follow up were included in the study. Twenty-six healthy subjects were included in the HC group in parallel. After the baseline visit, four healthy subjects discontinued their participation in the study for varied reasons. In total, 22 healthy subjects (18 females) completed the study. Demographic characteristics for the study groups are summarized in Table 1. There was no difference in sex distribution between the groups (Chi square *p* = 0.145); however, the BMS group was older than the HC (*p* = 0.016). Mean duration since MS diagnosis was 23.3 years at baseline visit. The highest EDSS score at the end of follow up was three in only one patient. Optic neuritis (ON) was found in seven patients at MS onset (38.9%), while one patient developed ON later. A total of 44.4% of BMS subjects had unilateral ON. At the onset of MS, seven patients (38.9%) had sensory loss; three patients (16.7%) had motor defect; and one patient (5.5%) presented with brain stem symptoms with vertigo and diplopia (Table 2). Notably, only four patients (22%) received treatment for MS symptoms prior to the study examinations.

The macular data from two BMS patients were excluded due to the development of epiretinal membrane and vitreomacular traction, respectively, at the follow up visit.

### 3.2. OCT Measures from BMS and HC Group

**Figure 1:**
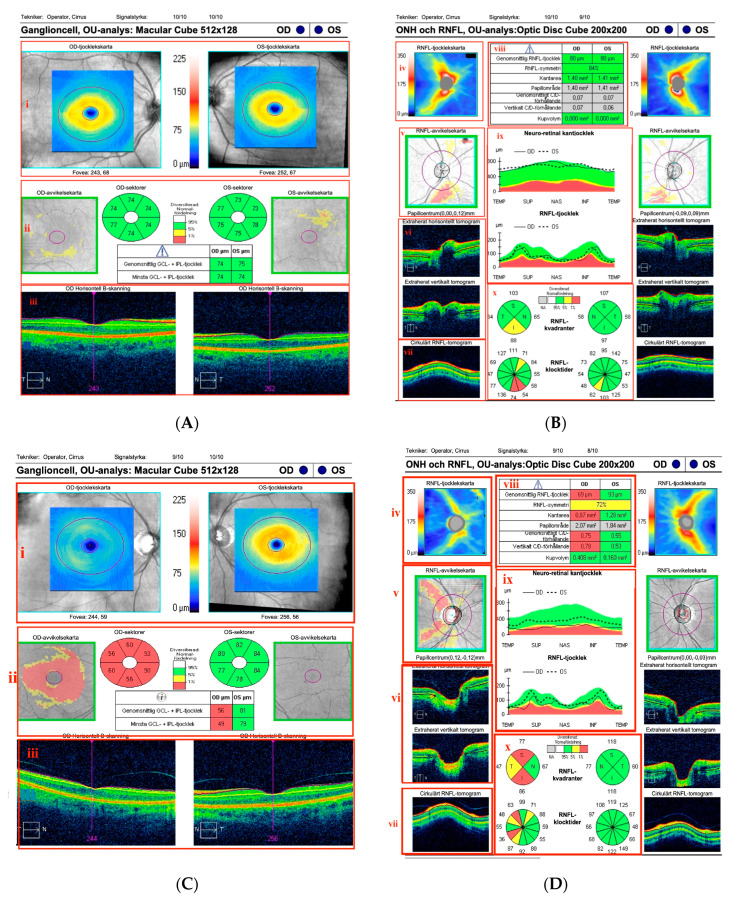
The OCT B-scans of the (**A**) macular GCL-IPL and (**B**) OCT of optic disc of a healthy subject showing normal thickness in both macular GCL-IPL and peripapillary RNFL in both eyes. (**C**) Macular OCT in a patient with 20 years duration of BMS and right-sided optic neuritis, EDSS = 0 and without treatment. The thickness map (i) shows clear thinned GCL-IPL layer in right eye (OD) compared to left eye (OS). The same result is shown in the thickness deviation map (ii), where the thinner layer is shown in red compared to green in OS with average thickness. (**D**) The optic disc report in the same patient shows a thinner average RNFL in OD vs. OS in the data table (viii). (iii) Horizontal and vertical B-scan centered on macula, (iv) RNFL thickness map, (v) RNFL thickness deviation map from normal thickness, (vi) horizontal and vertical RNFL thickness tomogram, (vii) circular RNFL thickness tomogram, (viii) data table with average RNFL thickness and optic disk parameters, (ix) neuro-retinal and RNFL thickness graph with normative data, and (x) thickness graph in quadrants and clock hour.

OCT parameters were collected from the OCT report for GCL-IPL and RNFL as shown in Figure 1 (A and B respectively). The OCT measures, including RNFL and GCL-IPL thinning rate values, were normally distributed (Shapiro–Wilk test > 0.05). The OCT data for the entire study population (all eyes) is summarized in Table 3. In general, the OCT measures in the BMS group were thinner than the HC group, both at baseline and at the final study visit. Except for the baseline AMT and final MV, the difference between the measures of both groups was significant even after adjustment for age.

Data from all eyes of both groups, including ON eyes, shows the mean thinning rate of RNFL/year between the groups was not significant (*p* = 0.562) and even after adjustment for age (*p* = 0.569). On the other hand, the rate of thinning/year of GCL-IPL was significant with a mean loss of 0.4 μm/year in the BMS group and gain of 0.02 µm/year in the HC group (*p* = 0.016 and *p* = 0.026 after age adjustment).

The OCT measures of the best eye per subject (excluding all ON eyes) summarized in Table 4 also indicated generally thinner measurements in the BMS group, but these were not statistically significant relative to controls except for the GCL-IPL layer (*p* < 0.001 at both baseline and final visit) even after adjustment for age alone or with both age and sex.

Changes in the RNFL layer were not different between groups before or after adjustment for age (*p* > 0.05). The rate of GCL-IPL layer change was not significantly different between groups before and after adjusting for age (*p* > 0.05) or for both age and sex. In the BMS group, the rates of RNFL and GCL-IPL change were not correlated with disease duration (r = −0.147, *p* = 0.560, C.I. 95% and r = −0.478, *p* = 0.061, C.I. 95%), respectively.

## 4. Discussion

The average thickness of GCL-IPL and RNFL (as shown in Figure 1) was considered for comparison instead of sectoral or clock hour thickness because of the normal variation in these measurements caused by retinal vessels distribution [23]. The diagnosis of BMS can be considered to be controversial [10] as there is no clear definition for this subgroup of MS [12,24]. The most accepted basis for the definition of BMS, however, is the low degree of disability experienced by the subject, even after a long-elapsed period from the first presentation of signs [25]. As new recommendations are nowadays applied for early treatment of MS patients with the use of different disease-modifying drugs that slow down disabilities and lower the EDSS [26] the diagnosis of BMS will no longer be possible on the same basis. The group of largely untreated BMS patients we examined here is unique; following current recommendations, it will be exceedingly difficult to identify this group with a benign course without treatment.

The patients included in the present study matched the BMS definition criteria according to Glad et al. and Portaccio et al. [4,5,6], and the most frequent presenting signs in the BMS group were sensory and optic neuritis, which is in line with what has been previously reported for BMS [27]. Fourteen of the BMS patients (78%) in this study remained untreated for MS, while the remaining four patients received first-line therapy appropriate to the benign course of the disease. The results reported here thus represent changes in the retinal thickness during the natural course of BMS, in contrast to patients with RR-MS, where most need to switch to second-line disease-modifying therapy [28].

The BMS group had thinner retinal layers in comparison to the HC group, even when only data from non-ON eyes was compared; the results, however, were not statistically significant for all measures. Our result agrees with other studies measuring the RNFL and GCL-IPL thickness in MS patients using OCT [20,29]. One possible explanation for a thinning retina could be a subclinical effect on the neuro-retinal layers, even in the absence of clinical or MRI signs of MS affecting the optic nerve, as evidence from postmortem studies has shown that optic nerve involvement may be present in 94–99% of MS cases [30].

GCL-IPL and RNFL thinning in MS in both ON-affected and non-ON eyes is well known [29,31,32,33,34,35]. In the present study, however, no correlation was noted between the thinning rate and disease duration in the non-ON BMS group, which may reflect the benign course of the disease.

Very few longitudinal studies have quantified RNFL and GCL-IPL; the rate of RNFL loss was similar in both MS and BMS, as reported by Galetta et al. [36], while no longitudinal data to our knowledge have been published concerning the GCL-IPL loss rate.

The rate of change of either RNFL or GCL-IPL between HC and BMS groups was not statistically significant. A small nonsignificant difference was present, however, and may partially be explained by the older age and overrepresentation of females in the BMS group as it has been reported that both older age and female sex are associated with thinner GCL and RNFL [37].

In a previous cross-sectional study on BMS, a clear difference was noted in the extrapolated thinning rate associated with disease duration both in the GCL-IPL and RNFL between BMS patients and patients with RR-MS [20]. In the present study, the rates of RNFL and GCL-IPL thinning in non-ON BMS were −0.20 ± 0.27 and −0.19 ± 0.15 μm/year, mean ± SE, respectively, in agreement with results from earlier extrapolated rates for RNFL and GCL-IPL, where thickness loss was −0.11 ± 0.27 and −0.24 ± 0.24 μm/year, respectively [20]. The earlier study also reported a thinning rate of 0.54 ± 0.24 μm/year for RNFL in RR-MS; in other studies, the reported rate of loss of RNFL thickness in MS differed from about 0.9 µm/year [38] to 2.4 µm/year [39].

Based on these data, we recommend considering a thinning rate of RNFL and GCL-IPL at 0.2 μm/year or less in patients with MS diagnosis as a biomarker of benign course. This biomarker could be combined with other parameters used in estimating benign MS course to possibly lead to earlier diagnosis, compared to the 10 or 15 years that is needed according to prior definitions. Those with greater loss rates as determined by longitudinal OCT examination could be followed up with further tests to possibly detect MS at an earlier stage.

The small population sample in the present study and the lack of a comparison group of RR-MS patients are limitations of the present study. A strength, however, is the presentation of data from a unique cohort of individuals with BMS with a natural MS course. To our knowledge, this is the first report of longitudinal measures of the retina in BMS patients, in particular the GCL-IPL layer.

In summary, the thinning of the RNFL and GCL-IPL layers in BMS has no correlation with disease duration and is statistically similar to a healthy population. These retinal thinning rates, measured over a period of 2 to 3 years, may therefore be considered as a biomarker for a benign course in patients with MS.

## Figures and Tables

**Table 1:** Demographic characteristics of the study population.

	HC	BMS	*p* Value
Subjects No. (Eyes)	22 (44)	18 (36)	
F/M	18/4	11/7	0.145 *
Age, years; mean (SD), range	51.7 (8), 32–65	60.6 (13.9), 37–82	0.016 ^#^
Disease duration, years; mean (range)	N/A	24.7 (11–40)	
EDSS score, mean (SD), range	N/A	1.27 (1.0), 0–3	
Total ON cases, n (%)	N/A	8 (44.4%)	
Follow-up time in months, mean (SD)	34.3 (16)	32.1 (10.9)	0.623

HC = healthy control group; BMS = benign multiple sclerosis; ON = optic neuritis; N/A = not applicable; and EDSS = expanded disability status scale. * *p* < 0.05 according to chi-Square. ^#^ According to independent *t*-test. (F/M) female/male, (SD) standard deviation.

**Table 2:** Clinical characteristics of the patients with BMS.

No.	Age *	Sex	Time Between Diagnosis and Baseline (y)	Symptom at Onset	EDSS at Final Visit	ON	Treatment	Date of Treatment Start
1	37	Male	11	sensory	0			
2	50	Male	20	sensory	0	Right **		
3	49	Male	18	leg weakness	0		Copaxone	2002
4	41	Female	28	ON right	1	Right	Interferon	2005
5	59	Female	23	sensory	2.5			
6	79	Male	22	Vertigo + diplopia	0			
7	73	Male	38	Walking severity, myelitis	2			
8	52	Male	21	sensory	0			
9	62	Female	35	hand weakness	2			
10	82	Female	28	ON right	2	Right		
11	39	Female	14	sensory	0		Interferon	2001
12	67	Female	35	ON Left	2	Left		
13	72	Female	40	ON Left	3	Left		
14	55	Male	18	ON right	1.5	Right	Interferon	2000
15	63	Female	16	ON Left	2	Left		
16	68	Female	29	ON right	1	Right		
17	62	Female	11	sensory	1			
18	80	Female	39	sensory	2.5			

* Age at baseline examination. EDSS = expanded disability status scale; ON = optic neuritis. ** Patient 2 developed right-sided ON 16 years after MS diagnosis.

**Table 3:** OCT measures of all eyes of BMS and HC groups.

	HC Mean (Range)	BMS Mean (Range)	*p* Value	*p* Adjusted for Age
Baseline				
RNFL (μm)	91.8 (75–109)	86.4 (66–112)	0.036	0.64
AMT (μm)	279 (248–308)	275.2 (244–296)	0.199	0.292
MV (mm^3^)	10.1 (9.2–11.1)	9.9 (8.8–10.7)	0.050	0.077
GCL-IPL (μm)	80.4 (69–90)	72.4 (55–85)	0.000	<0.001
Final study visit				
RNFL (μm)	91.3 (75–114)	85.5 (61–112)	0.032	0.027
AMT (μm)	280.4 (245–314)	271.9 (243–293)	0.025	0.047
MV (mm)	10.2 (9.1–11.3)	9.8 (8.8–10.6)	0.004	0.007
GCL-IPL (μm)	80.3 (69–91)	71.2 (54–84)	0.000	<0.001
Longitudinal data				
RNFL thickness change μm/year mean (SE)	−0.06 (0.26)	−0.29 (0.29)	0.562	0.569
GCL-IPL thickness change μm/year mean (SE)	0.02 (0.08)	−0.4 (0.19)	0.016	0.026

HC: healthy control group; BMS = benign multiple sclerosis; RNFL = peripapillary retinal nerve fiber layer; GCL-IPL = macular ganglion cell-inner plexiform layer; MV = mean volume; and AMT= average macular thickness.

**Table 4:** OCT measures of best eye from BMS and control group.

	HC, Mean (Range)	BMS, Mean (Range)	*p* (Value)	*p*. Adjusted for Age	*p* Adjusted for Age and Sex
Baseline					
RNFL (μm)	92.2 (79–109)	87.5 (68–112)	0.199	0.209	0.257
AMT (μm)	280.7 (254–308)	275.8 (244–292)	0.328	0.460	0.187
MV (mm3)	10.2 (9.2–11−1)	10 (8.8–10.5)	0.158	0.232	0.097
GCL-IPL (μm)	80.2 (69–90)	73.3 (60–85)	0.003	<0.001	<0.001
Final study visit					
RNFL (μm)	91.9 (76–110)	86.6 (66–112)	0.151	0.087	0.118
AMT (μm)	281.8 (252–314)	273 (244–291)	0.121	0.186	0.096
MV (mm)	10.2 (9.1–11.3)	9.9 (8.8–10.5)	0.051	0.078	0.056
GCL-IPL (μm)	80.2 (69–91)	72.5 (60–84)	0.002	<0.001	<0.001
Longitudinal data					
RNFL thickness change, μm/year mean (SE)	−0.05 (0.3)	−0.2 (0.27)	0.710	0.454	0.286
GCL-IPL thickness change, μm/year mean (SE)	−0.00 (0.11)	−0.19 (0.15)	0.292	0.573	0.067

BMS = benign multiple sclerosis; HC = healthy control group; RNFL = peripapillary retinal nerve fiber layer; GCL-IPL = macular ganglion cell-inner plexiform layer; MV = mean volume; and AMT = average macular thickness.

## Data Availability

Data are available upon request.
